# Internet Gaming Disorder in Adolescents: Personality, Psychopathology and Evaluation of a Psychological Intervention Combined With Parent Psychoeducation

**DOI:** 10.3389/fpsyg.2018.00787

**Published:** 2018-05-28

**Authors:** Vega González-Bueso, Juan J. Santamaría, Daniel Fernández, Laura Merino, Elena Montero, Susana Jiménez-Murcia, Amparo del Pino-Gutiérrez, Joan Ribas

**Affiliations:** ^1^Atención e Investigación en Socioadicciones (AIS), Mental Health and Addictions Network, Generalitat de Catalunya, Barcelona, Spain; ^2^Research and Development Unit, Parc Sanitari Sant Joan de Déu, Fundació Sant Joan de Déu, CIBERSAM, Sant Boi de Llobregat, Barcelona, Spain; ^3^School of Mathematics and Statistics, Victoria University of Wellington, Wellington, New Zealand; ^4^Pathological Gambling Unit, Department of Psychiatry, Bellvitge University Hospital-IDIBELL, Barcelona, Spain; ^5^Ciber Fisiopatología Obesidad y Nutrición, Instituto de Salud Carlos III, Madrid, Spain; ^6^Department of Clinical Sciences, School of Medicine and Health Sciences, University of Barcelona, Barcelona, Spain; ^7^Nursing Department of Mental Health, Public Health, Maternal and Child Health, Nursing School of the University of Barcelona, Barcelona, Spain

**Keywords:** adolescents, behavioral addiction, internet gaming disorder, parents, psychoeducational group, psychological treatment

## Abstract

Internet Gaming Disorder is an increasingly prevalent disorder, which can have severe consequences in affected young people and in their families. There is an urgent need to improve existing treatment programs; these are currently hampered by the lack of research in this area. It is necessary to more carefully define the symptomatic, psychosocial and personality characterization of these patients and the interaction between treatment and relevant variables. The objectives of this study were three: (1) to analyze the symptomatic and personality profiles of young patients with Internet Gaming Disorder in comparison with healthy controls; (2) to analyze the effectiveness of a cognitive behavioral treatment on reducing symptomatology; and (3) to compare the results of that treatment with or without the addition of a psychoeducational group offered to the parents. The final sample consisted of 30 patients consecutively admitted to a specialized mental health unit in Spain, and 30 healthy controls. The experimental group received individual cognitive-behavioral therapy. The experimental group was divided into two subgroups (*N* = 15), depending on the addition or not of a psychoeducational group for their parents (consecutively admitted). Scores on the Millon Adolescent Personality Inventory (MACI), the Symptom Checklist-Revised (SCL-90-R), the State-Trait Anxiety Index (STAI), and other clinical and psychopathological measures were recorded. The patients were re-assessed post treatment (except for the MACI questionnaire). Compared with healthy controls, patients did not differ in symptomatology at baseline, but scored significantly higher in the personality scales: Introversive and Inhibited, and in the expressed concerns scales: Identity Confusion, Self-Devaluation, and Peer Insecurity and scored significantly lower in the Histrionic and Egotistic scale. In the experimental group, pre-post changes differed statistically on SCL-90-R scales Hostility, Psychoticism, and Global Severity Index and on the diagnostic criteria for Internet Gaming Disorder, regardless of the addition of a psychoeducational group for parents. Pre-post changes did not differ between experimental subgroups. However, the subgroup without psychoeducation for parents presented statistically higher drop-out rates during treatment. The results of this study are based on a sample of patients seeking treatment related to problems with online gaming, therefore, they may be of value for similar patients.

## Introduction

In recent years, it has been recognized that addictions are not limited to behaviors generated by the uncontrolled use of substances (Echeburúa and Del Corral, 1999; Griffiths, 2000). There are seemingly harmless behavioral habits that, in certain circumstances, can become addictive and seriously interfere with the daily lives of those affected.

Due to growing evidence suggesting the existence of such behavioral addictions, the Diagnostic and Statistical Manual, DSM-5 (APA, 2013) included a new diagnostic category named Substance-Related and Addictive Disorders, in which is included the section Non-Substance-Related Disorders. Although this category includes only the diagnosis “Gambling Disorder” (F63.0), this is a step forward in the recognition of these disorders. Also, in Section III of the Manual (reserved for conditions that require further study), there has been included “Internet Gaming Disorder” (IGD). Likewise, “Gaming Disorder” has recently been included in the beta version of the International Classification of Diseases of the World Health Organization (ICD-11 Beta Draft–Mortality and Morbidity Statistics^1^). In this document the problem is defined as "a pattern of persistent or recurrent gaming behavior (“digital gaming” or “video-gaming”), which may be online (i.e., over the internet) or offline, manifested by (1) impaired control over gaming (e.g., onset, frequency, intensity, duration, termination, context); (2) increasing priority given to gaming to the extent that gaming takes precedence over other life interests and daily activities; and (3) continuation or escalation of gaming despite the occurrence of negative consequences. The behavior pattern is of sufficient severity to result in significant impairment in personal, family, social, educational, occupational or other important areas of functioning. The pattern of gaming behavior may be continuous or episodic and recurrent. The gaming behavior and other features are normally evident over a period of at least 12 months for a diagnosis to be assigned, although the required duration may be shortened if all diagnostic requirements are met and symptoms are severe.

Although these inclusions and the proposed diagnostic criteria help to establish and standardize the diagnostic tests for this type of disorders, there is an ongoing debate about the suitability of these criteria (Petry et al., 2014; Kuss et al., 2017) and about how to avoid pathologizing common behaviors (Billieux et al., 2017; Kardefelt-Winther et al., 2017; Tunney and James, 2017).

In the case of Internet and video games, research has shown that a proper use of these technologies may provide some benefits for its users (de Freitas and Griffiths, 2007; Hussain and Griffiths, 2008; Giner-Bartolomé et al., 2015). However, there is evidence that if used in excess it can become an addictive behavior (Griffiths, 2008). The impact of these behavioral addictions can be very serious, often impacting family and social relationships of the affected person or their academic or work responsibilities (Baer et al., 2011). These problems also seem to lead to comorbid psychological and health problems (e.g., depression, anxiety, social phobia) that accumulate and persist while the activity continues (Griffiths and Meredith, 2009).

In the clinical setting, new technology addiction is considered a multifactorial disorder from a variety of therapeutic approaches and perspectives: cognitive behavioral therapy (CBT), solution-focused therapy, interpersonal therapy or psychoeducation (Kuss and Griffiths, 2012). Several authors have explored the success of these treatments and shown that intervention with CBT is the most effective in reducing symptoms, and has better results in the short- and long-term (Young, 2007).

In this sense, research with clinical cases and healthy controls (including personality and psychopathology) can help to identify factors that explain issues related to the etiology of the Internet and video game addictions.

The scientific literature is scarce regarding the analysis of personality and psychopathology aspects associated with Internet Gaming Disorder (IGD). Most of the studies have focused on the analysis of healthy groups (adolescents, adults, and general population) through different types of surveys (i.e., online surveys, school surveys).

Some authors have found positive correlations between both Internet Addiction (IA) and Video Game Addiction (VA) and some personality traits: i.e., Psychoticism, Sensation Seeking, and Neuroticism (Cao and Su, 2007; Dalbudak et al., 2013), and negative correlations with Extraversion, Responsibility, Reward Dependence, Complacency, and Self-Directedness (Ko et al., 2007; van der Aa et al., 2009; Müller et al, 2013). Among personality traits, lower Responsibility has been identified as a risk factor associated with these disorders (Müller et al, 2013). Other authors have analyzed the relationship of personality traits predisposing to addiction (Griffiths, 2009) as Sensation Seeking, Self-Control, or Neuroticism, and problems related to videogame addiction. A relationship has been found between sensation seeking and VA, although these results are inconsistent (Wan and Chiou, 2006). Regarding self-control, it seems to have an influence on the incidence of the problem (Ng and Wiemer-Hastings, 2005): video games have a high capacity of immersion and this characteristic can lead to loss of control in some people. With respect to the third trait, Neuroticism, it has been observed that individuals with high scores on this personality scale are more likely to develop a VA (Huh and Bowman, 2007). Finally, in a study published in 2010 (Mehroof and Griffiths, 2010) the authors found that some personality characteristics, namely neuroticism, sensation seeking, traits and states of anxiety, were associated with the onset, development, and maintenance of a VA. In IGD specifically, certain personality factors, such as high neuroticism, high impulsivity, and high aggressiveness have consistently been found to be significant predictors of IGD (Collins et al., 2012; Billieux et al., 2015; Braun et al., 2016). Even so, most of the studies describing the relationship between personality and IGD are correlational and therefore unable to control for multiple comparisons, and/or are focused on the general population, thereby limiting their ability to establish causal links between specific personality traits and IGD (Gervasi et al., 2017).

Focusing on psychopathology, IGD has been associated with depression, ADHD, anxiety, and social phobia (Cole and Hooley, 2013; Hyun et al., 2015; Laconi et al., 2017; Wang et al., 2018). Specifically, depression seems to be the most common symptom associated with IGD in all age groups (adolescents, adults, and the general population). Other researchers have analyzed the psychiatric characteristics of heavy Internet users and have found a high prevalence of symptoms, including social anxiety, emotional problems, and cognitive deficits (Cao et al., 2007; Yen et al., 2008; Sun et al., 2009). Nevertheless, the relationship between these events remains unclear. First, it could be possible that a specific psychiatric problem leads one to develop IGD, second, that the associated problems and negative consequences of IGD will later develop into a psychiatric disorder, or third, that both problems share underlying biological, sociodemographic or psychological mechanisms, making people vulnerable to both pathologies (Dong et al., 2011). Less common are longitudinal studies. In this regard, in one study (Gentile et al., 2011), the authors concluded that the IGD can cause psychopathology, and that being diagnosed with VA or IA is related to the development of depression, anxiety, and social phobia symptoms.

One of the main problems regarding the efficacy of treatments for TA in adolescents is the lack of studies analyzing the issue and lack of standardized treatment protocols. Due to the lack of studies in this specific disorder, results regarding the efficacy of treatments are based on previous research focusing other behavioral addictions such as pathological gambling (López Viets and Miller, 1997; Petry and Armentano, 1999; Toneatto and Ladoceur, 2003), as well as recommendations of several experts (Widyanto and Griffiths, 2006; Peukert et al., 2010). The comparison between CBT and other psychological treatments for behavioral addictions has shown that CBT is more effective in reducing time spent in the problematic behavior and, as well, depressive symptoms.

Published studies specifically analyzing interventions for IA, VA, IGD, or other problems related with Internet use have included cognitive behavior therapy (CBT) (Young, 2007; Du et al., 2010; Li and Wang, 2013; Torres-Rodríguez et al., 2017), a CBT approach using virtual reality (Park et al., 2016), motivational interviewing (MI) combined with CBT (Orzack et al., 2006), psychological and/or counseling therapies using a self-devised treatment program (Su et al., 2011), family therapy (Han et al., 2012), pharmacological intervention combined with CBT (Han et al., 2010; Kim et al., 2012), and craving behavioral intervention (Zhang et al., 2016).

Evidence of the efficacy of CBT-based treatments in treating IGD is limited, due to methodological limitations and the lack of a critical mass of studies on any specific treatment. However, the results of the mentioned publications suggest that such interventions were useful in: (1) significantly reducing time spent gaming and other IGD symptoms; (2) reducing certain thoughts, cognitive distortions, and behaviors associated with compulsive video games use; (3) reducing comorbid symptomatology; and (4) increasing life satisfaction (Kim et al., 2012; Li and Wang, 2013; Park et al., 2016; Zhang et al., 2016).

Further, the results of a meta-analysis of treatment for IA (Winkler et al., 2013) indicate that this type of patient benefits more from individualized treatment, since characteristics such as social anxiety, social isolation, and lack of social competence (often present in these persons) can disturb the proper functioning of treatment groups. These authors also conclude that there is insufficient research on this topic.

In clinical settings, the experts usually detect in people affected with IGD the following: ignorance about the disorder characteristics, lack of family support, tense family relationships, lies, and arguments with the family (Griffiths and Meredith, 2009); these characteristics complicate some aspects of the treatment. Such dysfunctional family behaviors appear to be related to TA. Specifically, having positive support has been found to be negatively correlated with IA, while negative control and a low capacity for self-control is positively correlated with it. In addition, parental self-control has been found to play an indirect role in the behavior of parents and IA in their adolescent sons. Other authors have also found an association between family environment and online gaming addiction (Li et al., 2014; Hyun et al., 2015).

Few studies have focused on linking the psychoeducation of parents with patients' responses to treatment. Han et al. (2012) evaluated a brief 3-week family therapy intervention to change patterns of brain activation in response to affection and gaming cues in adolescents with IGD, and found that the intervention was linked to decreases in gaming time and IGD symptoms. Pallesen et al. (2015) investigated the efficacy of a manualized therapy including cognitive-behavioral therapy, short-term strategic family therapy, solution-focused therapy, and motivational interviewing, in 12 male adolescents with IGD and their mothers. The results showed a significant decrease in mother-reported IGD symptoms and a non-significant decrease in IGD symptoms in the adolescents. In addition, the importance of parental interest for the improvement of symptoms in adolescents with IA has been found (Lin et al., 2009; Young, 2009). These results suggest that there are some correlations between general parental behavior and patients' responses to treatment in IGD.

Summarizing, most studies examining aspects of personality or psychopathology related to the Internet and video game problems have been conducted in non-clinical settings and been based on surveys. There are fewer results focusing on clinical populations in controlled settings, with a diagnosis confirmed through structured interviews performed by experienced professionals. In addition, treatment potentials for these patients have not been sufficiently investigated.

In accordance with this need, the objectives of this study were as follows: (1) to evaluate the clinical, personality and socio-demographic characteristics of young patients diagnosed with IGD sample using a case-control design; (2) to investigate the effectiveness of a cognitive behavioral therapy in reducing symptomatology in patients with IGD; and (3) to analyze the effectiveness of the addition of a psychoeducational group therapy for these patients' parents.

## Methods

### Participants

The sample included 30 males (age between 12 and 21) diagnosed with an IGD who were consecutive referrals for assessment and outpatient treatment at the Behavioral Addiction Unit in the mental health center AIS-PRO JUVENTUD (Care and Research in Behavioral Addiction) (AIS), located at Barcelona, Spain; and 34 of their family members (mother, father or both). The control group included 30 healthy persons of similar age. Individuals attending the *Universitat Autonoma de Barcelona* area were asked to volunteer and recruited as healthy controls after signing an informed consent form.

Calculation of the required sample size was based on the standard deviations of questionnaire SCL-90-R. Thus, by setting an alpha risk of 0.05 and a beta risk of 0.20 in a two-sided test with a 10% estimated dropout rate, we required a sample size of 11 individuals in each group in order to detect a minimum expected difference between two groups of 0.2 units. We therefore decided to recruit 15 patients per group.

Individuals in the experimental group were excluded from the analyses if they: (1) had primary psychiatric or neurological disorders that could affect cognitive function (assessed through semi-structured, face-to-face, clinical interview in the case of the experimental group and by direct questions in the case of the healthy controls), (2) had a head injury with loss of consciousness for more than 2 min or a learning disorder, (3) used psychostimulants or drugs that could interfere with the treatment, (4) were older than 21 years or younger than twelve. For the control group of healthy persons, the exclusion criteria were: (1) had an Axis I (DSM-5) mental disorder, (2) be older than 21 years or younger than 12 years. No potential participants in either the experimental or control group were excluded on the basis of exclusion criteria 1, 2, or 3.

The Ethics Committee of CEIC Fundació Unió Catalana d'Hospitals (CEIC14/71) approved the study, and informed consent (signed document) was obtained from parents of adolescents under the age of 18 years and adolescents over the age of 18 years (and assent in adolescents under the age of 18 years).

### Instruments

#### Millon adolescent personality inventory (MACI) (Millon, 1993)

This self-administered personality test has 160 items, and measures a total of 31 scales: 12 Personality Patterns scales (Axis II) (i.e., Introversive, Inhibited, Doleful, Submissive, Histrionic, Egotistic, Unruly, Forceful, Conforming, Oppositional, Self-Demeaning, and Borderline Tendency), eight Expressed Concerns Scales (Identity Diffusion, Self-Devaluation, Body Disapproval, Sexual Discomfort, Peer Insecurity, Social Insensitivity, Family Discord, and Childhood Abuse), seven Clinical Syndrome Scales (Eating Dysfunctions, Substance Abuse Proneness, Delinquent Predisposition, Impulsive Propensity, Anxious Feelings, Depressive Affect, and Suicidal Tendency), three Modifying Indices which assess particular response styles (Disclosure, Desirability, and Debasement), and a Validity scale. The instrument has been translated to Spanish and validated in a Spanish population with a good internal consistency of 0.82 (Cronbach's alpha) (Aguirre, 2004).

#### Symptom checklist-90 items-revised (SCL-90-R) (Derogatis, 1990)

The SCL-90-R evaluates a range of psychological problems and psychopathological symptoms. The test contains 90 items and measures 9 primary symptom dimensions: Somatization, Obsession-Compulsion, Interpersonal Sensitivity, Depression, Anxiety, Hostility, Phobic Anxiety, Paranoid Ideation, and Psychoticism. It also includes three global indices: (1) a global severity index (GSI), designed to measure overall psychological distress, (2) a positive symptom distress index (PSDI), to measure the intensity of symptoms, and (3) a positive symptom total (PST). This scale has been translated to Spanish and validated in a Spanish population (Derogatis, 2002), and present a good internal consistency (Cronbach alpha = 0.75).

#### State-trait anxiety index (STAI) (Spielberger et al., 1970)

This is a self-report questionnaire that includes 40 items on a 4-point rating scale, measuring state anxiety (20 items) and trait anxiety (20 items). Scores range from 20 to 80 points. The state anxiety evaluates the current state of anxiety, using items that measure subjective feelings of apprehension, tension, nervousness, worry, and activation/arousal of the autonomic nervous system. The trait anxiety scale evaluates relatively stable aspects of “anxiety proneness,” including general states of calmness, confidence, and security. The STAI has been translated to Spanish and validated in the Spanish population with Cronbach's alpha coefficients ranging between 0.90 and 0.94 (Guillén-Riquelme and Buela-Casal, 2011).

#### Diagnostic questionnaires for video games, mobile phone or internet addiction (DQVMIA)

**Figure 1:**
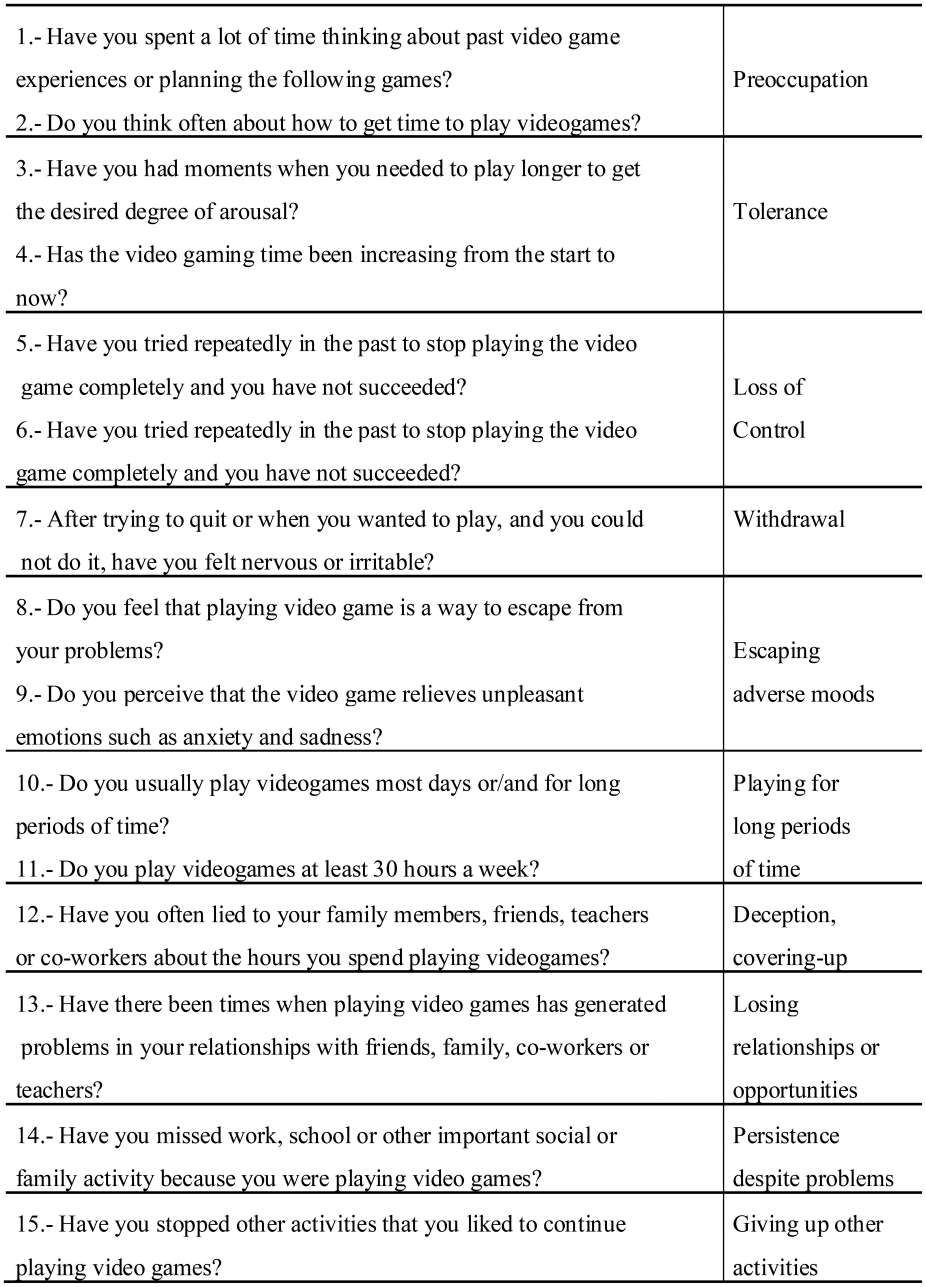
Questions included in the “Diagnostic questionnaires for Videogames, mobile phone or Internet addiction” (DQVMIA) and the correspondence with the DSM-5 diagnostic criteria for IGD.

This instrument is based on the Gambling Disorder Diagnostic Questionnaire (Stinchfield, 2003). It is a short questionnaire that examines the diagnostic criteria for Gambling Disorder (APA, 1994), adapted to the DSM-5 criteria for video games addiction. The Spanish version of the original questionnaire showed satisfactory internal consistency (α = 0.95; Jiménez-Murcia et al., 2009). This questionnaire covers all the diagnosis criteria proposed in the DSM-5 for Internet Gaming Disorder, i.e., preoccupation, tolerance, loss of control, withdrawal, escaping from adverse mental states, playing for long periods, deception/covering up, risking or losing relationships or opportunities because of the behavior, persistence of the behavior despite problems, and giving up other activities. The diagnoses were later reconfirmed by the results of the semi-structured face-to-face clinical interview described in the following section. Figure 1 shows the questions included in the questionnaire and its correspondence with the DSM-5 diagnostic criteria for IGD.

#### Other socio-demographic and clinical variables

Additional demographic, clinical, and social/family variables related to internet gaming were measured using a semi-structured face-to-face clinical interview.

### Procedure

We applied a quasi-experimental design, where the type of intervention was the independent variable. Experienced psychologists (more than 5 years of clinical experience in behavioral addictions) conducted the first face-to-face specific clinical interview and a functional analysis of IGD using a semi-structured clinical interview SCID-I (First et al., 1996), that includes questions regarding preoccupation, tolerance, loss of control, withdrawal, escaping from adverse mental states, playing for long periods, deception/covering up, risking or losing relationships or opportunities because of the behavior, persistence of the behavior despite problems, giving up other activities, and functional impairment (e.g., functional impairment in familial relationships, other social relationships, and academic achievement). In addition to a comprehensive clinical and psychological evaluation, including use of the instruments mentioned above, demographic data were also obtained at the beginning of therapy. This phase consisted of one session (with an average duration of 90 min). With regard to meeting the diagnostic criteria for IGD, the results obtained through the *Diagnostic questionnaires for video games, mobile phone, or Internet addiction (DQVMIA)* were compared post hoc with the results obtained through the face-to-face clinical interview, and only patients who met the DSM-5 criteria for IGD were included in our analysis (APA, 2013). Patients were also reassessed during the last therapy appointment (except for the MACI questionnaire).

The control group (CG) of healthy individuals (*N* = 30) completed the same tests battery (except for the semi-structured interview).

The persons in the Experimental Group (EG) (*N* = 30) were assigned (consecutively admitted) to individual cognitive behavioral therapy (all patients received the same therapy); their parents were either assigned or not assigned (consecutively admitted) to a psychoeducational group. This different condition (parents receiving psychoeducational group or not) was used to obtain two different subgroups:

Subgroup 1 (CBT) (EG1) or Subgroup 2 (CBT + PE for parents) (EG2).

#### Subgroup 1, EG1: individual cognitive behavioral therapy (CBT)

A total of 15 participants were assigned to this condition. An experienced psychologist applied cognitive behavioral therapy (CBT) consisted of 12 outpatient sessions of 45 min each, based on the treatment program manual for Gambling Disorder (Jiménez-Murcia et al., 2006). The frequency was as follows: weekly the first 4 sessions, biweekly the next 4 sessions and monthly the last four. This treatment was focused on the patients, and the goal was to train patients to implement CBT strategies to achieve control from using the Internet and video games. The general topics addressed included psycho-education regarding the disorder (i.e., its definition, phases, course, vulnerability factors, biopsychosocial models), stimulus control (time control, avoidance of risk situations, changing risky behaviors, etc.), cognitive restructuring focused on illusions of social success and magical thinking about studies, work and life success, response prevention (alternative and compensatory behaviors), reinforcement and self-reinforcement, skills training, and relapse-prevention techniques. The therapy was focused on the patient and a co-therapist was included in half on the sessions, which in all cases was a parent (father, mother or both) with the aim of strengthening, controlling and reinforcing some guidelines, and control outside the sessions.

#### Subgroup 2, EG2: individual cognitive behavioral therapy + psychoeducational group for their parents (CBT + PE for parents)

The patients assigned to this condition (*N* = 15) received the cognitive behavioral therapy described in the previous section. A co-therapist, which in all cases was a parent (father, mother, or both), was included in half of the sessions with the aim of strengthening, controlling, and reinforcing guidelines and control outside of the sessions.

**Figure 2:**
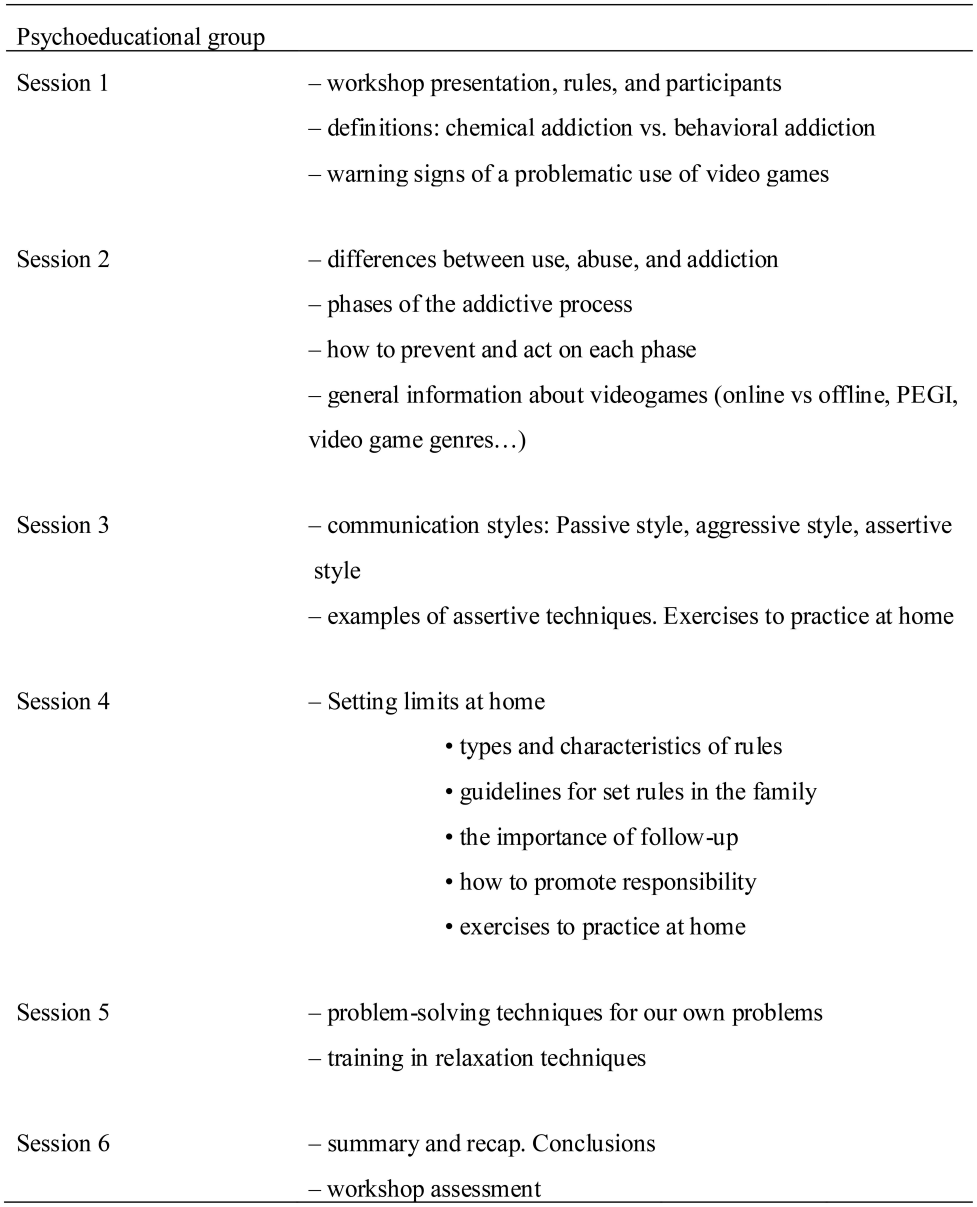
Description of the main concepts discussed in the psychoeducational group sessions.

Parallel to the first six sessions, a psychoeducational group (PE) regarding social and parental skills was provided to patients' parents (father, mother or both). This intervention was focused on the parents. During these sessions, only the parents were present. Only general concepts were discussed; individual problems related to each personal relative were not addressed. The goal was to help participants better understand the behavioral addictive disorders, improve communication skills and the ability to set limits and improve problem-solving skills. Figure 2 shows a description of the main concepts discussed in the different psychoeducational group sessions.

## Statistical analysis

All statistical analyses were carried out with the statistical software SPSS version 23 for Macintosh (IBM, 2013).

After applying a standard data exploration of the participants of both the experimental and the control groups, we did not observe any individual standing out from the others, and no outliers or missing information were detected. Therefore, we did not remove any person from the original data set and no transformation of the subscales was required.

The type of socio-demographic variables applied are continuous and binary. We compared the means and the proportions among the two experimental and control subgroups applying a one-way ANOVA and a Fisher's exact test, respectively. We used the Fisher's exact test instead of a Chi-squared test because the sample size in each group is small.

For each objective, we ran a different statistical method. The summary of the methods applied is as follows:

First, independent-samples tests were used to compare the experimental group (i.e., the two treatment conditions combined) and the control group on the clinical profiles (diagnostic criteria for IGD measures and global psychopathology) and on the personality profiles.

Second, a set of paired-samples tests was run to compare the effectiveness of the two treatment subgroups on SCL-90-R, STAI, and DQVMIA scores. The paired differences were computed between baseline and post-therapy in the combined experimental groups, given that all the patients in the experimental group received the same treatment conditions (during the psychoeducational therapy for parents, the patient was not present; nor were individual problems related to each personal relative addressed).

Third, analysis of covariance (ANCOVA) was used to compare the effectiveness of both treatment options (independent variable) while controlling for the SCL-90-R, STAI, and DQVMIA scores at baseline (because the probability of change in clinical measures is often strongly associated with initial values). In that manner, we controlled for an interaction effect between the scores of the two experimental subgroups (post) and the factor time (pre).

Cohen's *d* was calculated to measure the effect size for pairwise comparisons between groups (effect size was considered low at |*d*| < 0.50, moderate at |*d*| > 0.50, and high at |*d*| > 0.80). For the non-parametric version of the tests, i.e., Mann-Whitney and Wilcoxon Signed-Ranks tests, we calculated the effect size *r* = Z/√(N) proposed by Rosenthal (1994), where Z is the score and N is the total number of the samples (effect size was considered low at |*r*| < 0.10, moderate at |*r*| > 0.30, and high at |*r*| > 0.50).

For all tests, Levene's tests were carried out to check the assumption of equality of error variances. Moreover, Shapiro-Wilks' tests were also conducted to verify the normality of the observation because the sample size was small. When the normality assumption is rejected, non-parametric Wilcoxon Signed-Ranks and Mann-Whitney tests are used instead of independent-samples tests and the paired-samples tests, respectively. Additionally, we applied Bonferroni correction when we tested the effectiveness of the two treatment options, which provides an adjusted estimation of the group means, i.e., the estimated marginal means are adjusted for the effect of the controlled scores at baseline.

Finally, a survival analysis was conducted to compare the two experimental conditions in the event the patient withdrew the treatment where he is allocated. Time was measured as the number of sessions attended by the patients. Thus, survival distributions for both treatments were estimated by using a Kaplan-Meier estimator and the Log-Rank (Mantel, 1966), Breslow (Generalized Wilcoxon) (Breslow, 1970), and Tarone-Ware tests (Tarone and Ware, 1977) were computed to test their equality.

## Results

### Socio-demographic characteristics

The sample of the experimental group (EG) included 30 males diagnosed with an IGD. These persons were divided into two experimental subgroups including 15 individuals each in order to analyze the influence of the addition of a psychoeducational group for their parents. The control group (CG) of healthy subjects included 30 subjects. A summary of the socio-demographic characteristics can be found in Table 1.

**Table 1:** Socio-demographic and Clinical Variables of the Experimental Group.

	**Sample**		
	**EG1 (*N* = 15)**	**EG2 (*N* = 15)**	**CG (*N* = 30)**
Age (years); mean (SD)	15.5 (2.3)	16.1 (2.2)	17.4 (2.7)
Employed; %	6.7	0.0	0.0
Education level; % *Primary or less*	86.7	86.7	46.7
Duration of the problem (years); mean (SD)	1.8 (1.5)	1.9 (1.6)	–
Tobacco use, %	0.0	6.7	3.3
Drug use; %	0.0	6.7	0.0

*EG, Experimental group; CG, Control group*.

Statistical analysis comparing subgroup means for continuous socio-demographic variables and the association between subgroups and categorical socio-demographic variables, revealed no statistically significant differences for age, employment, education level, duration of the problem, or tobacco or drug use between the two experimental subgroups (CBT vs. CBT + PE) and the control group.

### Baseline clinical and personality characteristics of the experimental and controls groups

Table 2 gives the means and the results of the independent samples tests for all subscales to measure the differences in clinical variables between the experimental and control groups. The reported results were corrected by unequal error variances in the case that Levene's test was rejected.

**Table 2:** Comparison between experimental and healthy control groups of psychopathological and clinical outcomes at baseline.

	**EG Mean (SD)**	**CG Mean (SD)**	**Mean Difference (SD)**	**t**	**df**	**Sig. (2-tailed)−95%**	**Cohen effect size**
**INDEPENDENT SAMPLE TESTS**							
**SCL-90-R**							
Obsessive compulsive	0.97 (0.67)	1.02 (0.62)	−0.04 (0.16)	−0.26	58	0.795	−0.26
**STAI**							
State anxiety	18.00 (9.83)	16.10 (10.92)	7.86 (2.86)	0.71	58	0.482	0.71
Trait anxiety	21.17 (11.34)	18.63 (9.67)	8.31 (3.03)	0.93	58	0.356	0.93
			***z***			**Asymp. Sig. (2-tailed)**	**r effect size**
**MANN-WHITNEY TEST**							
**SCL-90-R**							
Somatization	0.60 (0.72)	0.54 (0.43)	−0.63			0.529	0.115
Interp. Sens.	0.91 (0.91)	0.76 (0.63)	−0.118			0.906	0.022
Depression	0.86 (0.88)	0.76 (0.76)	−0.059			0.953	0.011
Anxiety	0.60 (0.72)	0.63 (0.53)	−0.825			0.409	0.151
Hostility	1.01 (0.85)	0.80 (0.70)	−0.854			0.393	0.156
Phobia	0.35 (0.57)	0.20 (0.38)	−1.03			0.303	0.188
Paranoia	0.85 (0.92)	0.86 (0.65)	−0.541			0.588	0.099
Psychoticism	0.57 (0.65)	0.43 (0.49)	−0.582			0.56	0.106
Global severity	0.73 (0.65)	0.67 (0.48)	−0.081			0.935	0.015
**DQVMIA**							
Diagnostic criteria	6.20 (1.56)	2.07 (1.53)	−6.26			0.000^*^	1,143

*EG, Experimental Group; CG, Control Group; SD, Standard Deviation*;

* *significant result*.

No significant differences in psychopathology (measured with the SCL-90-R and the STAI questionnaires) were observed between EG and CG individuals at baseline. The mean scores of the two groups were under the mean clinical scores of the general population. However, significant differences were found when comparing scores of the Diagnostic Questionnaire for Videogames, Mobile Phone or Internet Addiction (DQVMIA) of both groups (*p* < 0.0005), as shown in Table 2.

Regarding personality characteristics, Table 3 shows the means and the results obtained from the MACI questionnaire, the EG showed significantly higher scores in the following Personality scales: Introversive and Inhibited, and in the following Expressed Concerns scales: Identity Confusion, Self-Devaluation, and Peer Insecurity, and significantly lower scores in the Histrionic and Egotistic scale.

**Table 3:** Comparison between experimental and healthy control groups of personality outcomes at baseline.

	**EG Mean (SD)**	**CG Mean (SD)**	**Mean Difference (SD)**	***t***	***df***	**Sig. (2-tailed)−95%**	**Cohen effect size**
**INDEPENDENT SAMPLE TESTS**							
**MACI**							
Introversive	26.44 (11.30)	18.58 (9.05)	7.86 (2.86)	2.74	49	0.008^*^	2.75
Inhibited	22.92 (13.19)	14.62 (7.63)	8.31 (3.03)	2.73	38.14	0.009^*^	2.74
Doleful	13.40 (10.57)	10.00 (10.67)	3.40 (2.97)	1.14	49	0.259	1.14
Submissive	46.44 (11.93)	45.73 (6.37)	0.71 (2.69)	0.26	36.33	0.794	0.26
Egotistic	29.00 (11.51)	37.04 (7.70)	−8.04 (2.75)	−2.92	41.70	0.006^*^	−2.92
Unruly	30.64 (10.91)	29.08 (12.29)	1.56 (3.26)	0.48	49	0.634	0.48
Conforming	44.24 (9.76)	46.46 (10.23)	−2.22 (2.80)	−0.79	49	0.432	−0.79
Oppositional	22.60 (10.19)	19.08 (9.21)	3.52 (2.72)	1.30	49	0.201	1.30
Borderline tendency	14.24 (9.38)	10.19 (7.43)	4.05 (2.37)	1.71	49	0.093	1.71
Identity Confusion	17.92 (9.26)	12.62 (6.70)	5.31 (2.26)	2.35	49	0.023^*^	2.35
Self-Devaluation	24.36 (17.75)	14.92 (10.29)	9.44 (4.08)	2.31	38.20	0.026^*^	2.31
Sexual Discomfort	27.88 (7.57)	27.31 (7.45)	0.57 (2.10)	0.27	49	0.787	0.27
Peer Insecurity	10.88 (7.08)	7.23 (3.85)	3.65 (1.61)	2.27	36.72	0.029^*^	2.27
Family Discord	16.92 (5.21)	16.69 (7.24)	0.23 (1.78)	0.13	49	0.898	0.13
Childhood Abuse	7.68 (5.61)	5.04 (3.43)	2.64 (1.31)	2.02	39.49	0.06	2.02
Subst. Abuse Proneness	14.64 (9.20)	16.96 (11.77)	−2.32 (2.97)	−0.78	49	0.438	−0.78
Delinquent Predisposition	23.48 (6.99)	26.08 (7.30)	−2.60 (2.00)	−1.30	49	0.201	−1.30
Impulsive Propensity	15.72 (7.61)	15.35 (6.69)	0.37 (2.00)	0.19	49	0.853	0.19
Anxious Feelings	30.08 (8.36)	29.85 (6.53)	0.23 (2.10)	0.11	49	0.912	0.11
			***z***			**Asymp. Sig. (2-tailed)**	
**MANN-WHITNEY TEST**							
**MACI**							
Histrionic	35.12 (11.19)	43.15 (8.94)	−2.38			0.017^*^	0.43
Forceful	11.92 (8.32)	8.54 (5.08)	−1.35			0.177	0.24
Self-Demeaning	25.16 (18.20)	15.31 (12.58)	−1.82			0.069	0.33
Body Disapproval	9.24 (8.13)	4.92 (4.71)	−1.684			0.092	0.31
Social Insensitivity	23.80 (7.58)	26.92 (8.38)	−1.37			0.171	0.25
Eating Dysfunctions	8.96 (8.38)	5.81 (4.93)	−0.87			0.384	0.16
Depressive Affect	17.64 (12.18)	11.77 (8.89)	−1.774			0.076	0.32
Suicidal Tendency	7.96 (7.62)	4.62 (6.52)	−1.969			0.051	0.36

*EG, Experimental Group; CG, Control Group; SD, Standard Deviation*.

* *Significant result*.

### Individual cognitive-behavioral therapy outcomes

Table 4 reports the results of the paired samples tests for the experimental group as a whole (*N* = 30).

**Table 4:** Pre-post comparisons for SCL-90-R, STAI, and DQVMIA mean scores for the experimental group (*N* = 30).

	**Paired differences Mean (SD)**	***t***	***df***	**Sig. (2-tailed)−95%**	**Cohen effect size**
**PAIRED SAMPLES TEST**					
**SCL-90-R**					
Somatization	−0.07 (0.28)	−1.06	19	0.30	−0.237
Obsessive compulsive	−0.22 (0.50)	−1.96	19	0.06	−0.437
Interp. Sens.	−0.15 (0.60)	−1.16	19	0.28	−0.249
Depression	−0.08 (0.40)	−0.86	19	0.40	−0.192
Anxiety	−0.12 (0.37)	−1.38	19	0.18	−0.308
Hostility	−0.33 (0.65)	−2.25	19	0.04^*^	−0.504
Paranoia	−0.13 (0.45)	−1.32	19	0.20	−0.296
Global severity	−0.14 (0.29)	−2.13	19	0.04^*^	−0.476
**STAI**					
State anxiety	−0.60 (8.00)	−0.34	19	0.74	−0.075
Trait anxiety	0.40 (8.07)	0.22	19	0.83	0.050
	***z***			**Asymp. Sig. (2-tailed)**	
**WILCOXON SIGNED-RANKS TEST**					
**SCL-90-R**					
Phobia	−0.06			0.95	0.011
Psychoticism	−2.37			0.02^*^	0.433
**DQVMIA**					
Total score	−3.84			0.00^*^	0.701

*SD, Standard Deviation*;

* *Significant result*.

Pre- and post-scores of the whole sample did not statistically differ for the STAI questionnaire. In contrast, pre-post scores differed statistically on SCL − 90 -R scales, Hostility (*t* = −2.25, *p* = 0.036), Psychoticism (*z* = −2.37, *p* = 0.02) and Global Severity Index (*t* = −2.13, *p* = 0.047), and on the total score of the DQVMIA (*z* = −3.84, *p* < 0.000).

### Pre-post changes in psychopathology based on the addition of a psychoeducational group for parents

In order to test whether incorporating an additional parents' psychoeducational group improves results of the treatment regarding psychopathology, an ANCOVA regressions controlled by the baseline values (pre) was performed. When comparing the pre-post mean scores between the two experimental subgroups (CBT + PE vs. CBT) regarding the different scores on the SCL-90-R, STAI, and DQVMIA questionnaires, no differences were found at 0.05 significance level.

### Survival analysis for drop-out comparison based on the addition of a psychoeducational group for parents

**Figure 3:**
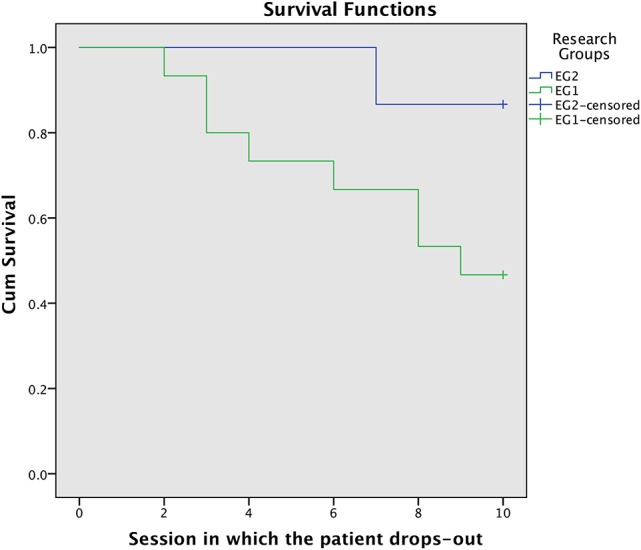
Dropout rate (y-axis) of each experimental subgroup in each treatment session.

Total dropout rate in the experimental group as a whole was 33.33%. In the experimental subgroup with parental psychoeducation the dropout rate was 13.33%, and in the experimental group without parental psychoeducation was 53.33%. Survival analysis concludes that the drop-out rate is statistically different in both experimental subgroups according to the test statistics for equality of survival distributions: Log-rank test (χ^2^ = 5.478, *p*-value = 0.019), Breslow test (χ^2^ = 5.472, *p*-value = 0.019), and Tarone-Ware test (χ^2^ = 5.483, *p*-value = 0.019). Additionally, Figure 3 shows the drop-out rate of each experimental subgroup in each treatment session. The graphical difference for both drop-out distributions is easy to observe.

## Discussion

This study investigated the effectiveness of a cognitive behavioral treatment (with and without an addition of a psychoeducational group for parents) in a sample of patients with IGD and assessed the therapy outcome by means of survival analyses of these two conditions. In addition, we explored the psychopathological and personality characteristics of the participants compared with a healthy control group.

As reported in earlier studies (Weiser, 2000; Tsitsika et al., 2013), males tend to use the Internet for activities related to entertainment and leisure. This preference may be mediated by age; in our sample all the patients with IGD were male adolescents, and their main problem behavior were online games.

Compared with healthy subjects, the patients showed higher scores in several personality characteristics (i.e., Introversive, Inhibited, and Histrionic, Identity Confusion, Self-Devaluation, and Peer Insecurity) and lower scores in Egotistic personality scale. Regarding the efficacy of the CBT applied, after the treatment the patients reported less general symptomatology (SCL-90-R scales, Hostility, Psychoticism, and Global Severity Index), and less diagnostic criteria for IGD. Finally, the addition of a psychoeducational group for parents seemed not to help to reduce symptomatology, but fewer dropouts in the CBT + PE groups were observed.

### Personality characteristics and psychopathological symptoms

Patients, when compared with the group of healthy subjects, scored higher on introversion, were more concerned about their identity, valued themselves less and were more insecure in relationships with peers. Our findings seem to be in agreement with reports showing that patients with a technological addiction (TA) have a reduced extraversion (Ko et al., 2009; van der Aa et al., 2009; Müller et al, 2013). These concerns about themselves and others, combined with introversive traits, could lead these adolescents to avoid face-to-face activities. They also might try to cover these social needs in the digital world through online games, where one can interact with others remotely and in a superficial way (Griffiths and Meredith, 2009). As for the other personality characteristics found, high scores on inhibition tend to show subjects as anxious and apprehensive, expecting life to be painful. Thus, people scoring high on this scale tend to avoid situations evaluated as potentially aversive and tend to fall into isolation. People with high histrionic levels tend to be talkative, socially charming and often emotionally expressive. The statistically lower scores on these scale in the experimental group, combined with the other personality factors, could favor the abuse of online video games due to the intrinsic characteristics of these technologies: the isolation from the real world and its ability to facilitate immersion in the activity (Griffiths and Meredith, 2009).

Regarding the clinical symptoms, in our sample, no significant differences in overall psychopathology among the group of patients and healthy controls were found, except for the IGD criteria. There may be several hypotheses for this result. Although some authors have found correlations between IGD in adolescents and depression, anxiety and ADHD symptoms (Baer et al., 2011; King and Delfabbro, 2016), others have found small effect sizes or non-relationships (Van Rooij et al., 2011; King et al., 2013; Vadlin et al., 2016). Analyzing the mean scores on the SCL-90-R of our sample, we found that the patient's scales did not reach clinical levels. In the same line of the longitudinal study performed by Gentile and co-workers on problematic use of videogames (Gentile et al., 2011), those adolescents who became and stayed pathological gamers ended up with increased levels of depression, anxiety, and social phobia after 2 years of duration, demonstrating that gaming predicts other mental health disorders longitudinally, rather than simply being correlated with them. The average problem duration in our patients was less than 2 years. It is therefore possible that the negative consequences their problem was causing had not yet affected their mental health.

Moreover, some authors focusing on other behavioral addictions (Gambling Disorder) have found that younger adults, as opposed to older ones, do not experience psychological discomfort apart from the symptoms of the addiction (González-Ibáñez et al., 2005). The authors conclude that probably older gamblers have experienced the negative consequences of the disorder for a longer period, and this has led them to develop comorbid psychopathology. It is possible that the psychological symptoms associated with IGD, as reported in some studies analyzing healthy population with Internet or videogames addiction (Cao and Su, 2007; Berner et al., 2014), are due to lack of control in the duration of the problem and/or primary and previous mental disorder variables.

Finally, each online video game usually has an associated players' community. Therefore, it is possible to establish online relationships, which are often informal and superficial, with people with similar interests. The personality characteristics of these patients could help them to feel themselves more comfortable with these online social relationships and build their lives around it, thereby alleviating psychological distress.

In any case, our results support the hypothesis that although adolescents with IGD can use games as a coping mechanism, IGD is not simply a symptom of other psychological problems.

### Cognitive behavioral therapy outcomes

One of the main problems regarding the efficacy of treatments for IGD is the lack of both sufficient studies reporting results and standardized treatment protocols. As authors use different approaches to treat the problem, data comparison is usually difficult and unreliable.

Based on results obtained in other behavioral addictions such as Gambling Disorder (López Viets and Miller, 1997; Petry and Armentano, 1999; Toneatto and Ladoceur, 2003), and the results of papers analyzing IGD (Kim et al., 2012; Li and Wang, 2013; Park et al., 2016; Zhang et al., 2016) we would expect a cognitive behavioral therapy approach to be useful in reducing the direct symptoms related to the addiction and other comorbid symptomatology. The results of our study partially support this view. Regardless of whether or not a psychoeducational group for relatives was included in the treatment, we found a significant reduction in the applied diagnostic criteria in our patients, corroborating previous studies showing that the symptoms associated with TA can be successfully treated with CBT (Thorens et al., 2012; Winkler et al., 2013). Patients also showed a significant reduction in Hostility, Psychoticism, and Global Severity index as measured by the SCL-90-R. Nevertheless, we have to take into account the fact that scores on these psychopathological scales were higher, although not significantly so, in the patient sample than in the healthy controls sample.

The reduction in the Hostility scale (interpreted as thoughts, feelings and characteristic actions of the negative emotional state of rage or anger) and Psychoticism scale (interpreted as a conduct of social isolation), could be of great importance as these two variables have been described as two of the predictors of the onset of TA. This is especially true of Hostility, which has been described as the most significant predictor of Internet addiction in male adolescents in a two-year follow-up study (Ko et al., 2009). Hence, this reduction could help patients to become more resistant to developing TA in the future.

### Outcomes and dropouts comparison based on the addition of a psychoeducational group for parents

Our study shows that patients in the CBT group with an associated psychoeducational group for parents have lower dropout rates.

Few studies analyzing CBT treatment of Internet addiction-related problems have reported on dropout rates, making comparisons with our results difficult. The dropout rates that we could find in the recent literature include 6.67% in IGD patients treated with a combination of CBT and electroacupuncture (Zhu et al., 2012), 24.6% in IA patients treated with cognitive behavioral therapy and motivational interview (Thorens et al., 2014), and 80% in patients with problematic, Internet-enabled sexual behavior treated with a psycho-educational program combining psychodynamic and cognitive-behavioral therapy (Orzack et al., 2006).

None of these articles provided information about the factors associated with dropping out. In the treatment of similar disorders, such as Pathological Gambling (PG), some authors have described the role of certain personality traits (i.e., neuroticism, impulsivity, and sensation-seeking) (Leblond et al., 2003; Álvarez-Moya et al., 2011), age of onset (Melville et al., 2007), and motivation (Gómez-Peña et al., 2011).

Our results suggest that the addition of a psychoeducational group for parents may not mitigate the symptomatology of the patient but may increase therapy adherence and reduce the dropout rate. In accordance with findings for PG, one possible explanation for our reduced dropout rate in this subgroup could be related to the external motivation of the patient. The use of CBT to treat addictions (McKay, 2007) and specifically TA (Young, 2007) requires an effort from the patient, therefore motivation is a crucial aspect in achieving results. In this regard, parental interest, a greater involvement, understanding of the disorder and family-related abilities of the closest relatives of the patient could facilitate some treatment aspects. A number of studies have revealed a direct relationship between general family functioning and Internet addiction (Şenormanci et al., 2014; Ko et al., 2015); their results show that better general family functioning reduces the chances of becoming an Internet addict. Additionally, some authors (Young, 1999) have suggested that family groups focused on educating the parents on understanding the addiction, and improving the communication about problems in the family, can be an important part of such therapies. They further suggest that a strong sense of family support may enable the patient to recover from Internet addiction. Thus, the inclusion of a parent (father or mother) during the therapy sessions, where only problems directly related with the IGD are treated, and these crucial aspects are not focused may be not sufficient to complement the individual treatment.

### Limitations

Limitations of this study include: (1) The results of this study are based on a small sample. It is therefore difficult to generalize the conclusions, although they may be of value for similar patient profiles, i.e., patients seeking treatment in relation to IGD. In addition, considering we used a clinical sample evaluated in a controlled setting, there may be reason to anticipate our findings could be confirmed in future studies. (2) Measures used in this study are based on self-administered questionnaires, although it should be noted that patients were supervised by a trained psychologist to ensure the highest quality of data collection. (3) The lack of a control group of patients who did not receive any treatment (there is no waiting list in our unit) or other treatment options (e.g., group therapy), has not allowed us to perform other comparisons. (4) The short-term nature of the study did not allow to us to examine their middle- to long-term effects. (5) The drop-out rate was higher than expected (10%). In average, the power for all the tests was 75% instead of the planned 80%.

## Author contributions

VG-B and JS contributed to design the work. VG-B, JS, LM, and EM collaborated in the collection and the interpretation of the data. DF carried out the statistical analysis. JS, VG-B, and DF drafted the study. All authors revised the article critically for important intellectual content, commented on and approved the final manuscript. All authors are accountable for all aspects of the work.

### Conflict of interest statement

The authors declare that the research was conducted in the absence of any commercial or financial relationships that could be construed as a potential conflict of interest.

## Footnotes

^1^ICD-11 Beta Draft - Mortality and Morbidity Statistics Available at: https://icd.who.int/dev11/l-m/en [Accessed November 17, 2017].
